# Different Methods for Modelling Severe Hypoglycaemic Events: Implications for Effectiveness, Costs and Health Utilities

**DOI:** 10.1007/s40273-018-0612-y

**Published:** 2018-02-14

**Authors:** Edna Keeney, Dalia Dawoud, Sofia Dias

**Affiliations:** 1 Bristol Medical School, University of Bristol, Canynge Hall, 39 Whatley Road, Bristol, BS8 2PS UK; 2 National Guideline Centre, Royal College of Physicians, London, UK; 3 Faculty of Pharmacy, Cairo University, Cairo, Egypt

## Abstract

**Background:**

Clinical trials report severe hypoglycaemic events as the number of patients with at least one event out of the total randomised or number of events for a given total exposure. Different network meta-analysis models have been used to analyse these different data types.

**Objective:**

This aim of this article was to establish the impact of using the different models on effectiveness, costs and health utility estimates.

**Methods:**

We analysed a dataset used in a recent network meta-analysis of severe hypoglycaemic events conducted to inform National Institute for Health and Care Excellence recommendations regarding basal insulin choice for patients with type 1 diabetes mellitus. We fitted a model with a binomial likelihood reporting odds ratios (using a logit link) or hazard ratios (complementary log-log link), a model with a Poisson likelihood reporting hazard ratios and a shared-parameter model combining different types of data. We compared the results in terms of relative effects and resulting cost and disutility estimates.

**Results:**

Relative treatment effects are similar regardless of which model or scale is used. Differences were seen in the probability of having an event on the baseline treatment with the logit model giving a baseline probability of 0.07, the complementary log-log 0.17 and the Poisson 0.29. These translate into differences of up to £110 in the yearly cost of a hypoglycaemic event and 0.004 in disutility.

**Conclusion:**

While choice of network meta-analysis model does not have a meaningful impact on relative effects for this outcome, care should be taken to ensure that the baseline probabilities used in an economic model are accurate to avoid misrepresenting costs and effects.

**Electronic supplementary material:**

The online version of this article (10.1007/s40273-018-0612-y) contains supplementary material, which is available to authorized users.

## Key Points

**Table:** 

The method used to model severe hypoglycaemic events can have an impact on the estimated probability of having an event. This article shows that some statistical modelling methods give a lower probability while others give higher.
As probabilities are the inputs used in economic models, a lower or higher probability can have a substantial impact on the costs and utilities estimated.
It is important to ensure that probabilities of severe hypoglycaemic events are accurately calculated to avoid misrepresenting the cost effectiveness of treatments.

## Background

Severe hypoglycaemia can occur in people with diabetes mellitus who take insulin and other anti-diabetic treatments. Clinical trials use variable definitions for severe hypoglycaemia but it is generally defined as having low blood glucose levels that require assistance from another person to treat and is classed as a diabetic emergency that can lead to seizures, coma or death [1]. Trials also report hypoglycaemic events in different forms. Some report the number of patients who experienced at least one event out of the total number randomised (risk) and others report the number of events for a given total exposure (rate). This makes combining results from different trials to conduct a meta-analysis based on aggregate data alone a challenging task and poses a question about whether there are advantages of using one outcome over another.

In the context of economic modelling, the costs and quality of life (QoL) losses associated with these events should be taken into account. Hence, measuring the risk of having a hypoglycaemic event as opposed to the rate/number of events, could lead to underestimating the costs and QoL losses associated with these events as only the costs and disutilties associated with one event will be considered. Results of economic analyses, in terms of the most cost-effective treatment, often hinge on small differences in costs and utilities and it is therefore important to represent these accurately. The issues highlighted in this article are therefore relevant not only in the case of severe hypoglycaemic events but in any situation where repeated events in the same patient are possible.

Published Bayesian network meta-analyses (NMAs) of trials of treatments to prevent hypoglycaemic events have used either the binomial with logit link [2] for data reported as the risk of an event, or Poisson with log link [3] for data reported as the rate of events. These models estimate relative treatment effects as odds ratios or hazard ratios, respectively. Another model that would be considered suitable for such data is the binomial model with a complementary log-log (clog-log) link [4]. This model assumes an underlying Poisson rate of events, but can be used when data are reported as the risk of an event after a certain time period. In the first part of this article, we use the data from the systematic review and NMA conducted to inform recommendations regarding basal insulin choice for patients with type 1 diabetes from the 2015 National Institute for Health and Care Excellence (NICE) guideline [5] to compare the relative effectiveness results from the three suggested models. These results will then be compared with those from a shared-parameter model [4], which has the advantage of being able to combine both risk and rate data.

The second part of this article considers the impact of using the binomial with logit or clog-log links and Poisson models on conducting a meta-analysis of studies to inform baseline effects. The baseline probability of an event is a person’s risk, or probability, of having an event when using the reference treatment, often placebo. The baseline probability of an event can be informed by using values from a cohort study or local database, using the reference treatment arm from a particular trial, which represents the UK population, or by performing a meta-analysis of relevant treatment arms from all or a subset of included trials. In this study, we conduct Bayesian meta-analyses of all the reference treatment arms of the studies that compared the reference treatment, glargine (once), to any of the remaining insulin regimens [6].

Absolute probabilities of an event on each treatment are the inputs most commonly used in economic models. As these are calculated by applying the relative effects of each treatment to the baseline effect, different assumptions about this baseline effect can have a substantial impact on the costs and QoL outputs of economic models, even when relative effects are unchanged. We assess the impact different data types and modelling strategies could have on costs and QoL estimates.

## Methods

### Relative Effects

**Fig. 1:**
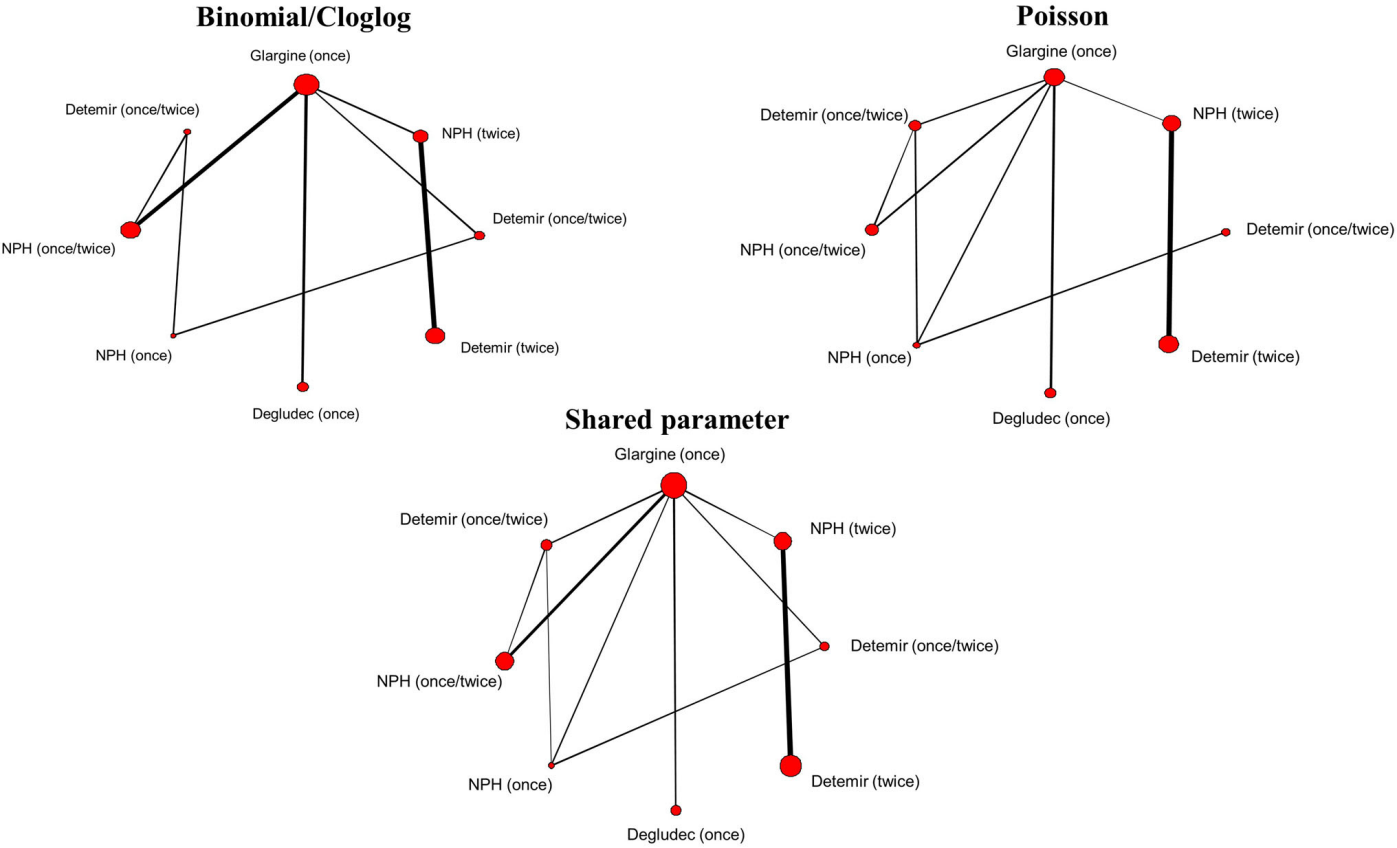
Network plots of studies included in each analysis. The lines connecting each pair of interventions represent a direct comparison in one or more randomised controlled trials. The width of the lines is proportional to the number of trials directly comparing each pair of interventions. The size of each node is proportional to the number of participants randomised to that treatment (sample size)

An NMA uses all the available evidence, both direct and indirect, to produce estimates of the relative effects of each treatment compared with every other in a network, even if some pairs of treatments have not been directly compared [4, 7–10] Network meta-analyses were conducted to simultaneously compare eight insulin regimens: insulin detemir once daily, insulin detemir twice daily, degludec once daily, insulin neutral protamine Hagedorn (NPH) once daily, insulin NPH twice daily, insulin NPH four times daily, insulin detemir once or twice daily and insulin NPH once or twice daily to the reference treatment, insulin glargine once daily. The network of evidence for each model is shown in Fig. 1 and the data used in each model are given in Appendix A of the Electronic Supplementary Material (ESM).

All analyses were conducted within a Bayesian framework using WinBUGS 1.4.3, Boca Raton, FL [11]. The binomial and Poisson models were based on the approach and code provided by the NICE Decision Support Unit [4]. The code for the shared-parameter model was adapted from the code provided by the NICE Decision Support Unit [4]. The code used in each model is provided in Appendix B of the ESM.

Twenty studies were available for meta-analysis. Twelve studies reported data as both the risk of having a hypoglycaemic event and the rate of events, while four studies only reported the risk and four only reported the rate. All studies met the inclusion criteria for the NICE guideline on type 1 diabetes [5] and were deemed eligible to be compared in a NMA based on the similarity of the populations included. In terms of baseline glycosylated haemoglobin, the percentage was similar between most of the studies (between 7.5 and 8.5%) and none were below 7%. The baseline age was also similar between studies (most were between 35 and 45 years of age). Further details on the inclusion and exclusion criteria are provided in the NICE guideline [5].

The 16 studies that reported risk data were synthesised using two alternative models. The first model adopts a binomial likelihood with a logit link function, and generates output on a log-odds scale. Relative treatment effects are reported as posterior median odds ratios and 95% credible intervals (CrIs). This model assumes linearity of effects on the logit scale and does not consider follow-up time of the trials, assuming that differences in follow-up time have no effect on how likely a patient is to have a first severe hypoglycaemic event. This model will henceforth be referred to as the *logit model.*

The follow-up time in the included trials varied from 4 weeks to 2 years. The second model, assumed a constant rate of events, to estimate the probability of events occurring over time. Again, a binomial likelihood is assumed, but a clog-log link function is used, which results in outputs on a log-hazard scale. Relative treatment effects are reported as posterior median hazard ratios and 95% CrIs. We will refer to this as the *clog-log model*.

For the 16 studies that reported rates over person-time, a *Poisson* model with a log link function was used to estimate the probability of events occurring over time. This model also produces outputs on a log-hazard scale. Relative treatment effects are reported as posterior median hazard ratios and 95% CrIs. This model, like the clog-log model, assumes that in each arm of each trial the hazard is constant over the follow-up period and also that the events are independent; thus, a person who has already had an event is no more likely to have a subsequent event than a person who has not yet had their first event.

An advantage of taking a Bayesian approach to estimation is that it is straightforward to extend to shared-parameter models where different trials report outcomes in different formats but from a common underlying model. As both rate and risk data can be synthesised on a log-hazard scale, it is possible to combine both in a shared-parameter model using a binomial likelihood with a clog-log link function for the risk data and a Poisson likelihood with a log link function for the rate data [4]. This assumes that, regardless of how the data are reported, the incidence of events has the characteristics of a homogeneous Poisson process. This model also assumes that after a patient has their first event, the rate of subsequent events does not change. A model of this type was estimated to combine the risk and rate data. This model could incorporate all 20 studies. For studies that reported both risk and rate data, rate data were given preference. Relative treatment effects are reported as posterior median hazard ratios and 95% CrIs.

Both fixed- and random-effects models were estimated. The goodness of fit of each model to the data was measured using the posterior mean of the residual deviance, which is a measure of the magnitude of the difference between the observed data and the model predictions for those data. Smaller values are preferred, and in a well-fitting model, the posterior mean residual deviance should be close to the number of data points [12]. The Deviance information criterion (DIC), which is equal to the sum of the posterior mean of the residual deviance and the effective number of parameters and penalises model fit with model complexity, was used as the basis for model comparison with lower values being favoured [12]. Differences of less than 3 were not considered meaningful and the simpler model was selected. Model selection was also based on the posterior median between-study heterogeneity and its CrI. Consistency between the different sources of indirect and direct evidence was explored statistically by comparing the fit of a model assuming consistency with a model that allowed for inconsistency (also known as an unrelated treatment-effect model). In this type of model, each of the comparisons for which evidence is available represents a separate unrelated basic parameter to be estimated [13]. If the inconsistency model had the smallest posterior mean residual deviance, heterogeneity or DIC value, then this would indicate potential inconsistency in the data.

Results are reported in terms of relative effects of each treatment compared with the reference treatment, glargine (once), for each of the models analysed. The posterior median of the ranking of each treatment (and 95% CrIs) is also reported, with the convention that the lower the rank the better the treatment.

### Absolute Probabilities

We also report the absolute probability of an event on each treatment, using the relative effects from each model. To estimate this, we needed to make an assumption about the absolute effect of the reference treatment, in this case, glargine (once), and apply this to the relative effects. Glargine (once) was chosen as the reference treatment as it was in the centre of the network of evidence and was directly compared to the most other treatments, increasing the stability of its relative effect estimates.

We derived the probability of an event on glargine (once) by conducting a Bayesian meta-analysis of all the glargine (once) arms of the studies that compared glargine (once) with any of the remaining insulin regimens. This was also done within a Bayesian framework using WinBUGS 1.4.3 and based on the approach described in Dias et al. [6]. The code is provided in Appendix B of the ESM.

There were eight studies reporting risk data that compared glargine (once) with any of the remaining insulin regimens (Appendix A of the ESM). A separate analysis of these arms was first carried out on the log-odds scale to apply to the relative effects from the logit model. The baseline probability was also estimated using the clog-log link (log-hazard ration scale) through a meta-analysis of the glargine (once) arms of the risk data.

The baseline rate of severe/major hypoglycaemia, defined here as the number of severe/major hypoglycaemic events per person-year of follow-up when using insulin glargine (once), was also calculated using a Poisson likelihood. There were seven studies in the rate data that compared glargine (once) to any of the remaining insulin regimens (Appendix A of the ESM).

Synthesising risk data using a binomial likelihood gives the absolute probability of having a hypoglycaemic event in 1 year, whereas synthesising rate data using a Poisson likelihood gives an absolute rate of events in 1 year. To make the results comparable, the absolute rates from the Poisson model were transformed into probabilities using the formula:

$$p \, = \, 1 \, {-}\exp \left( { - rt} \right)$$

where *p* is the probability, *r* is the rate and *t* is the time period of interest (in this case, 1 year) [14]. This conversion assumes constant rates.

Both fixed- and random-effects models were estimated for each model and the goodness of fit of each model to the data was again measured by comparing the posterior mean of the summed residual deviance, the DIC and the posterior mean between-study heterogeneity. The results from the three models and their effect on the absolute probabilities are explored below.

### Estimating Costs and Disutilities

To demonstrate the difference that the use of each of these models could have on costs and disutilities, we used a cost of £333 per severe hypoglycaemic event, estimated from Hammer et al. [15], and a utility decrement of − 0.012 for anyone experiencing a severe hypoglycaemic event (taken from NICE clinical guideline NG17 on type 1 diabetes [5]), and multiplied these by the absolute probabilities of having an event calculated from the three models.

## Results

### Model Fit

For the logit and clog-log models, the fit of the fixed-effects model was comparable to the random-effects model with the fixed-effects models having a higher residual deviance but a lower DIC. However, for comparison with the Poisson model (where random effects are required), we assess consistency and present results using random effects for all. No meaningful differences were observed in posterior mean residual deviance or DIC values when comparing the random-effects consistency and inconsistency models, suggesting that there was no evidence of inconsistency. The goodness-of-fit and model selection statistics for the models are reported in Appendix C of the ESM.

### Relative Effects

**Fig. 2:**
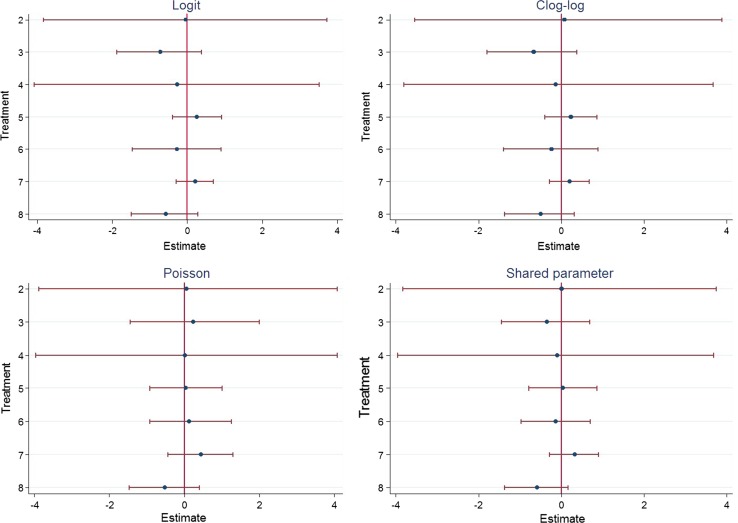
Mean differences in hypoglycaemic events. Negative values mean that the treatment reduces hypoglycaemic events compared with Glargine (once). Treatment legend: 2. Neutral protamine Hagedorn [NPH] (twice); 3. Detemir (once); 4. Detemir (twice); 5. Degludec (once); 6. NPH (once); 7. NPH (once/twice); 8. Detemir (once/twice). *Clog-log* complementary log-log

Reported results are based on the random-effects NMA models, assuming consistency. Results in terms of relative effects were relatively consistent across all models. Figure 2 shows the relative effects compared with glargine (once) when using each model, with the logit model on the log odds ratio scale and the other models on the log hazard ratio scale. The figure shows that there is very little difference between treatments in the reduction of events and that choice of model has little effect on this. One exception is for detemir (once), which seems more likely to increase hypoglycaemic events compared with glargine (once) in the Poisson model but is likely to decrease events in all other models. This suggests that there is a higher chance of having repeated events on detemir (once), which is not captured by the data included in the other models. However, overall for this dataset, choice of model scale has little impact on results and no firm conclusions can be drawn on which is the best treatment based on efficacy alone, owing to the uncertainty in the results.

Table 1 lists the treatments in terms of median rank and 95% CrIs. In the logit and clog-log models, detemir (once) has the highest median rank (second, CrI first to sixth) followed by detemir (once/twice) [third, CrI first to sixth]. Detemir (once) is replaced by detemir (once/twice) as the highest-ranking treatment in the Poisson and shared-parameter models. Neutral protamine Hagedorn (once/twice) is the lowest ranked treatment in all models. However, the wide CrIs around these ranks reflect the considerable uncertainty in the relative treatment effects and no firm conclusions on which is the best treatment can be drawn (Fig. 2).

**Table 1:** Posterior median rank and 95% credible intervals (CrIs)

Treatment	Logit and clog-log		Poisson		Shared parameter	
	Median rank	95% CrIs	Median rank	95% CrIs	Median rank	95% CrIs
Detemir (once)	2	(1–6)	5	(1–8)	3	(1–8)
Detemir (once/twice)	3	(1–6)	2	(1–6)	2	(1–6)
Detemir (twice)	4	(1–8)	4	(1–8)	4	(1–8)
NPH (once)	4	(1–8)	5	(1–8)	4	(1–8)
Glargine (once)	5	(2–7)	4	(2–7)	5	(2–7)
NPH (twice)	5	(1–8)	5	(1–8)	5	(1–8)
Degludec (once)	*6*	(2–8)	4	(1–8)	5	(1–8)
NPH (once/twice)	6	(3–8)	6	(2–8)	7	(3–8)

*clog-log* complementary log-log, *NPH* Neutral protamine Hagedorn

### Absolute Probabilities

The probability of having a hypoglycaemic event on the reference treatment was calculated separately using a logit, clog-log and Poisson model [6]. In each case, the random-effects model was a better fit to the data.

The single-arm meta-analysis of the eight studies comparing glargine (once) and reporting risk data using a logit model, produced a mean baseline probability of severe/major hypoglycaemic events of 0.07 (95% CrI 0.04–0.13) when using glargine (once). The meta-analysis using the same studies but with a clog-log model produced a mean baseline probability of severe/major hypoglycaemic events at 1 year of 0.17 (95% CrI 0.06–0.34). The analysis of the seven studies comparing glargine (once) and reporting rate data, using a Poisson likelihood, produced a mean baseline rate of severe/major hypoglycaemic events of 0.38 events per person-year (95% CrI 0.07–1.21). When this was transformed into a probability at 1 year, the baseline probability of having an event when using glargine (once) was 0.29 (95% CrI 0.07–0.7).

The difference in the baseline probabilities estimated is the result of the different time periods assumed. The logit model assumes the probability is the same for any time period but reflects the probability of an event over the study follow-ups in the contributing data. These have an average follow-up time of 5 months. The clog-log model gives a probability of an event over a 1-year period; thus, it follows that this will be larger. The Poisson model reflects the possibility of multiple events, which again leads to a higher probability. The Poisson model also has slightly different data included, owing to not all studies reporting both risk and rate data, although five out of the seven studies are common across all three models (Appendix A of the ESM).

Table 2 shows the absolute probabilities of events when the probabilities of having an event on glargine (once) from each of the baseline meta-analysis models are applied to the relative effects from the corresponding NMA model. This table shows that despite the relative effects being very similar (Fig. 2), the difference in baseline probabilities causes the absolute probabilities to differ considerably across models.

**Table 2:** Absolute probabilities of having a hypoglycaemic event (at 1 year)

Treatment	Absolute probability (logit)		Absolute probability (clog-log)		Absolute probability (Poisson)	
	Mean	95% CrIs	Mean	95% CrIs	Mean	95% CrIs
Detemir (once)	0.04	(0.01–0.11)	0.10	(0.02–0.29)	0.37	(0.04–0.97)
Detemir (once/twice)	0.04	(0.01–0.1)	0.11	(0.03–0.29)	0.2	(0.03–0.61)
NPH (once)	0.06	(0.01–0.17)	0.15	(0.03–0.43)	0.33	(0.05–0.86)
Glargine (once)	0.07	(0.04–0.12)	0.17	(0.07–0.34)	0.29	(0.07–0.7)
NPH (once/twice)	0.08	(0.04–0.16)	0.20	(0.07–0.43)	0.4	(0.08–0.91)
Degludec (once)	0.09	(0.03–0.18)	0.21	(0.07–0.47)	0.31	(0.05–0.81)
Detemir (twice)	0.12	(0–0.71)	0.26	(0–1)	0.38	(0–1)
NPH (twice)	0.14	(0–0.75)	0.29	(0–1)	0.39	(0–1)

*clog-log* complementary log-log, *CrIs* credible intervals, *NPH* Neutral protamine Hagedorn

### Expected Costs and Disutilities

Table 3 shows the expected costs from each of the models and Table 4 shows the expected disutilities. Although the ranking of costs and disutilities from lowest to highest is similar across the models, there is considerable difference in the estimated values. The expected cost of detemir (once), for example, varies from £13.29 per year when the absolute probabilities from the logit model are used, to £123.28 when the values from the Poisson model are used. The difference in disutilties is less apparent as the figures are small but the change in magnitude across the models is the same.

**Table 3:** Expected costs (£)

Treatment	Expected cost (logit)		Expected cost (clog-log)		Expected cost (Poisson)	
	Mean	95% CrIs	Mean	95% CrIs	Mean	95% CrIs
Detemir (once)	13.29	(2.97–36.83)	34.21	(6.88–97.52)	123.8	(13.21–323)
Detemir (once/twice)	14.41	(4.17–34.16)	38.16	(9.81–97.26)	66.91	(10.31–201.7)
NPH (once)	20.42	(4.38–57.71)	51.11	(10.14–145)	110.4	(18.24–287.6)
Glargine (once)	22.65	(11.76–39.04)	56.14	(22.35–112.6)	95.59	(22.34–233.5)
NPH (once/twice)	28.08	(12.17–53.85)	68.36	(24.27–144.5)	134.6	(27.28–302.8)
Degludec (once)	29.63	(11.53–61.19)	71.1	(23.44–156.8)	102.7	(18.24–287.6)
Detemir (twice)	41.67	(0.35–237.9)	87.82	(1.13–332.8)	126.7	(1.43–333)
NPH (twice)	47.37	(0.44–251.1)	97.82	(1.43–333)	128.3	(1.55–333)

*clog-log* complementary log-log, *CrIs* credible intervals, *NPH* Neutral protamine Hagedorn

**Table 4:** Expected disutilities

Treatment	Expected disutility (logit)		Expected disutility (clog-log)		Expected disutility (Poisson)	
	Mean	95% CrIs	Mean	95% CrIs	Mean	95% CrIs
Detemir (once)	0.000	(− 0.001 to 0)	− 0.001	(− 0.004 to 0)	− 0.004	(− 0.012 to 0)
Glargine (once)	− 0.001	(− 0.001 to 0)	− 0.002	(− 0.004 to − 0.001)	− 0.003	(− 0.008 to − 0.001)
Detemir (twice)	− 0.001	(− 0.009 to 0)	− 0.003	(− 0.012 to 0)	− 0.005	(− 0.012 to 0)
Degludec (once)	− 0.001	(− 0.002 to 0)	− 0.003	(− 0.006 to − 0.001)	− 0.004	(− 0.01 to − 0.001)
NPH (once)	− 0.001	(− 0.002 to 0)	− 0.002	(− 0.005 to 0)	− 0.004	(− 0.01 to − 0.001)
NPH (once/twice)	− 0.001	(− 0.002 to 0)	− 0.002	(− 0.005 to − 0.001)	− 0.005	(− 0.011 to − 0.001)
Detemir (once/twice)	− 0.001	(− 0.001 to 0)	− 0.001	(− 0.004 to 0)	− 0.002	(− 0.007 to 0)
NPH (twice)	− 0.002	(− 0.009 to 0)	− 0.004	(− 0.012 to 0)	− 0.005	(− 0.012 to 0)

*clog-log* complementary log-log, *CrIs* credible intervals, *NPH* Neutral protamine Hagedorn

## Discussion

This article found that, in the context of treatments to reduce hypoglycaemic events, choice of scale for the relative treatment effects does not have a significant impact on efficacy results. Although the Poisson model is expected to be the most appropriate for modelling these data as it takes into account different follow-up times and repeated events, because severe hypoglycaemic events are rare and occur at a constant rate, modelling relative treatment effects using odds ratios rather than hazard ratios still captures the differences between treatments.

However, as trials currently report severe hypoglycaemic events in different forms, and may continue to do so, the use of a shared-parameter model is recommended as this allows the incorporation of both risk and rate data; making use of *all* available data. Although incorporating both types of data did not make a considerable difference to results in this example, this may change in a different example.

Where extra care should be taken is in ensuring that the baseline probability of an event used in an economic model is realistic and accurate to avoid over or underestimation of the costs and effects. This article has demonstrated that choice of model and scale can have a significant effect on this baseline probability, with differences of between 0.07 and 0.29 found depending on the model and data used. It has also shown that when this baseline probability is applied to the relative effects from the corresponding model, significant differences can be seen in cost and utility estimates. It is feasible that these differences could lead to changes in the decision as to which treatment is most cost effective when the absolute probabilities are used in cost-effectiveness models.

The different assumptions made by the models and the data used explain the differences in results. The Poisson and clog-log model allow for a constant rate of events over time. These models are preferable to the logit model, which estimates a probability of at least one event that is unchanged over time. In addition, the Poisson data allow for more than one event per person, which allows a better estimation of the rate of events, when multiple events can occur (as is the case here).

The sensitivity of cost-effectiveness results to the hypoglycaemic event rate has been highlighted in several studies. Gschwend et al. [16], who used the IMS CORE Diabetes Model to estimate the cost effectiveness of insulin detemir compared with NPH insulin in patients with type 1 diabetes using data from a 2-year randomised controlled trial, showed that results were highly sensitive to the event rate used with small differences in the rate causing detemir to lose dominancy over NPH.

McEwan et al. [17] describe the Cardiff Type 1 Diabetes Model and report the quality-adjusted life-year impact of changes in the rate of hypoglycaemic events. The default baseline rate of a severe hypoglycaemic event used in the model is based on the probability of self-reporting a severe hypoglycaemic event of 0.46 in patients with type 1 diabetes from an observational study carried out by the UK Hypoglycaemia Study Group [18]. Modifying hypoglycaemia frequency by − 10, − 20 or −30% resulted in changes to discounted quality-adjusted life-years of + 0.05, + 0.11 and + 0.17, respectively, and modifying hypoglycaemia frequency by + 10, + 20 or +30% resulted in changes to discounted quality-adjusted life-years of − 0.05, − 0.09 and − 0.13. This emphasises the importance of accurately reflecting the probability of hypoglycaemic events in economic models.

The difficulty of calculating the cost of hypoglycaemic events based on studies reporting events in different forms has also been highlighted by Jonsson et al. [19] who used a cost-of-illness approach, based on an incidence methodology, to estimate the cost of hypoglycaemia in patients with type 2 diabetes. The incidence of hypoglycaemia was based on findings from several published studies, some reporting the number of events per person-years and some reporting the percentage of patients experiencing an event. They stated that the risk of hypoglycaemic events may therefore be slightly underestimated but did not adjust for this. Our analysis shows that this underestimation could potentially have a significant impact on cost-effectiveness results.

A strength of this work is that the dataset used to compare the different models in this paper is the same as that used in the NICE guideline on type 1 diabetes. The systematic review carried out to inform this guideline found 20 studies comparing basal insulin regimens but reporting different outcome measures. For the guideline, it was decided to only use the 16 studies that reported rate data and analyse these using a Poisson model. This article has demonstrated the difference in results had they opted to use the risk data, as has been done in a previous NMA [2].

The limitation of using these 20 studies reporting different outcome measures is that slightly different number of trials, and hence data, have gone into each analysis. The results are therefore not completely comparable and this may be a source of heterogeneity. It is evident from the network plots in Fig. 1 which direct comparisons are included in the logit/clog-log and Poisson models and which are missing. An alternative method would be to only include the 12 studies reporting both risk and rate data in each analysis and compare results on this basis, although this would mean excluding data, which will reduce the power of the analysis, making the networks even more uncertain. The strength of the shared-parameter model proposed here is that it can incorporate all the data and we recommend using this model wherever possible. There is, however, a relatively small number of studies per comparison, even in the shared-parameter model, leading to sparse networks. This means that the analyses have low statistical power to detect differences between the included treatments and it is not possible to make a recommendation regarding the optimal basal insulin regimen based solely on these analyses.

Another issue to consider is the uncertainty in the literature as to the exact definition of severe hypoglycaemic events, which may lead to between-trial heterogeneity in analyses such as these. The NICE guideline defined severe hypoglycaemia as ‘a blood glucose level that is sufficiently low to cause a reduced level of function in an individual such that they are unable to self-manage a hypoglycaemia episode and require help from another individual to achieve normoglycaemia.’ Most of the studies included in these analyses were in line with this definition, referring to the person needing assistance from another to treat, although some went into more detail, specifying the exact blood glucose level cut-off. Standardisation in this regard is recommended to reduce uncertainty in future analyses.

A further limitation is the lack of access to patient-level data. Insulin regimens that reduce complications such as severe hypoglycaemia are likely to also improve the stability of glycosylated haemoglobin, a reflection of average plasma glucose levels over the medium term (2–3 months), and also to reduce the occurrence of long-term complications and premature mortality [20–22] All of these are important factors when choosing the most appropriate treatment, but it is not possible to disentangle these effects with aggregate data.

In terms of future research, a further model comparison that could be made is with the negative binomial model, which is useful in the case of overdispersed data, i.e. when the observed variance is greater than expected under a Poisson model. The negative binomial is more flexible than the Poisson distribution, although it includes the Poisson distribution as a limiting case. We have not yet seen this model implemented in a meta-analysis context and because we only have aggregate data on the number of events per person-year, we did not have reason to suspect over-dispersion; thus, we chose to use the simpler Poisson model, which fitted well. Because of the flexibility of WinBUGS, the model could be adapted to include this more flexible approach, if required [11].

In addition, we were unable to access the IMS Core Diabetes Model [23] (which was used to assess cost effectiveness in the NICE guideline) and re-estimate the cost effectiveness using the different probabilities obtained. This is also an area for future research, as it would determine whether the differences in probabilities of severe hypoglycaemic events identified would in fact impact on the treatment decision.

In general, we recommend using the model that best fits with the underlying data-generating process. In the case of severe hypoglycaemic events, the Poisson model for the rate of events per person-year is the most appropriate. Based on the results from this model and the cost and disutilites of an event alone, detemir (once/twice) is the best intervention. The findings of this study suggest that in the case of severe hypoglycaemic events, when repeated events are taken into account, the baseline probability of having an event on glargine (once) is 0.29 but with wide CrIs of 0.07–0.7. Further exploration should be given to this baseline probability to ensure that it is accurate for use in economic models.

## Conclusion

This work has shown that choice of model and scale has little impact on relative effectiveness or on the ranking of basal insulin regimens from best to worst. No firm conclusions can be drawn with regard to the best basal insulin regimen for preventing severe hypoglycaemic events owing to the sparsity of the data and uncertainty in the networks.

However, we have shown that, despite this uncertainty in terms of relative effects, it is possible for incorrect conclusions to be drawn in terms of costs and disutilites, as absolute probabilities of events can easily be underestimated if the baseline probability does not take repeated events into account. This is particularly important in health economic models where small differences can have a considerable impact on results. Care should therefore be taken to choose an appropriate outcome measure when synthesising data on repeated events for use in an economic model.

## Electronic supplementary material

Below is the link to the electronic supplementary material.

Supplementary material 1 (DOCX 27 kb)

Supplementary material 2 (DOCX 21 kb)

Supplementary material 3 (DOCX 13 kb)
