# Correction to: Different Methods for Modelling Severe Hypoglycaemic Events: Implications for Effectiveness, Costs and Health Utilities

**DOI:** 10.1007/s40273-019-00778-5

**Published:** 2019-03-09

**Authors:** Edna Keeney, Dalia Dawoud, Sofia Dias

**Affiliations:** 1 Bristol Medical School, University of Bristol, Canynge Hall, 39 Whatley Road, Bristol, BS8 2PS UK; 2 National Guideline Centre, Royal College of Physicians, London, UK; 3 Faculty of Pharmacy, Cairo University, Cairo, Egypt

## Correction to: PharmacoEconomics (2018) 36:523–532 10.1007/s40273-018-0612-y

The Open Access license, which previously read:

**Open Access** This article is distributed under the terms of the Creative Commons Attribution-NonCommercial 4.0International License (http://creativecommons.org/licenses/by-nc/4.0/), which permits any noncommercial use, distribution,and reproduction in any medium, provided you give appropriate credit to the original author(s) and the source, provide a link to the Creative Commons license, and indicate if changes were made.

Should read:

**Open Access** This article is distributed under the terms of the Creative Commons Attribution 4.0 International License (http://creativecommons.org/licenses/by/4.0/), which permits unrestricted use, distribution, and reproduction in any medium, provided you give appropriate credit to the original author(s) and the source, provide a link to the Creative Commons license, and indicate if changes were made.

The original article has been corrected.

