# Supplementary material for: Different Methods for Modelling Severe Hypoglycaemic Events: Implications for Effectiveness, Costs and Health Utilities
Source: Pharmacoeconomics. 2018 Feb 14;36(5):523–32. doi: 10.1007/s40273-018-0612-y (PMC5906516; doi:10.1007/s40273-018-0612-y)
Supplement: Supplementary file 1 — Supplementary material 1 (DOCX 27 kb) [file 40273_2018_612_MOESM1_ESM.docx]

1. Data used in each model
   1. Studies used in analyses of relative effects

| Study | Binomial/Cloglog | Poisson | Shared parameter model |
| --- | --- | --- | --- |
|  |  |  |  |
| CHATTERJEE 2007^1^ | 🗸 | 🗸 | 🗸 |
| HERMANSEN 2004^2^ | 🗸 | 🗸 | 🗸 |
| HOME 2004^3^ | 🗸 | 🗸 | 🗸 |
| STANDL 2004^4^ | 🗸 | 🗸 | 🗸 |
| VAGUE 2003^5^ | 🗸 | 🗸 | 🗸 |
| LEEUW 2005^6^ | 🗸 |  | 🗸 |
| RENARD 2011^7^ | 🗸 |  | 🗸 |
| HELLER 2012^8^ | 🗸 | 🗸 | 🗸 |
| MATHIEU 2013^9^ | 🗸 | 🗸 | 🗸 |
| RASKIN 2000^10^ | 🗸 | 🗸 | 🗸 |
| RATNER 2000^11^ | 🗸 | 🗸 | 🗸 |
| HOME 2005^12^ | 🗸 |  | 🗸 |
| PIEBER 2000^13^ | 🗸 |  | 🗸 |
| RUSSELL JONES 2004^14^ | 🗸 | 🗸 | 🗸 |
| HERMANSEN 2001^15^ | 🗸 | 🗸 | 🗸 |
| BARTLEY 2008^16^ | 🗸 | 🗸 | 🗸 |
| KOLENDORF 2006^17^ |  | 🗸 | 🗸 |
| PIEBER 2005^18^ |  | 🗸 | 🗸 |
| FULCHER 2005^19^ |  | 🗸 | 🗸 |
| HELLER 2009^20^ |  | 🗸 | 🗸 |

- 1. Studies used in analyses of baseline probability

| Study | Binomial/Cloglog | Poisson |
| --- | --- | --- |
|  |  |  |
| CHATTERJEE 2007^1^ | 🗸 | 🗸 |
| RENARD 2011^7^ | 🗸 |  |
| HELLER 2012^8^ | 🗸 | 🗸 |
| MATHIEU 2013^9^ | 🗸 | 🗸 |
| RASKIN 2000^10^ | 🗸 | 🗸 |
| RATNER 2000^11^ | 🗸 | 🗸 |
| HOME 2005^12^ | 🗸 |  |
| PIEBER 2000^13^ | 🗸 |  |
| FULCHER 2005^19^ |  | 🗸 |
| HELLER 2009^20^ |  | 🗸 |

- 1. Study data for severe/major hypoglycaemia- Binomial/cloglog models

| Study | Comparator 1 | Comparator 2 | Comparator 1 | | Comparator 2 | | Trial follow-up time (Years)^*^ |
| --- | --- | --- | --- | --- | --- | --- | --- |
|  |  |  | N | NR | N | NR |  |
| CHATTERJEE 2007^1^ | NPH  (Twice) | Glargine  (Once) | 1 | 58 | 1 | 58 | 0.308 |
| HERMANSEN 2004^2^ | NPH  (Twice) | Detemir  (Twice) | 18 | 297 | 19 | 298 | 0.346 |
| HOME 2004^3^ | NPH  (Twice) | Detemir  (Twice) | 10 | 132 | 11 | 139 | 0.308 |
| STANDL 2004^4^ | NPH  (Twice) | Detemir  (Twice) | 14 | 135 | 18 | 154 | 1 |
| VAGUE 2003^5^ | NPH  (Twice) | Detemir  (Twice) | 21 | 146 | 24 | 301 | 1 |
| LEEUW 2005^6^ | NPH  (Twice) | Detemir  (Twice) | 21 | 99 | 30 | 216 | 0.5 |
| RENARD 2011^7^ | Glargine  (Once) | Detemir  (Once) | 10 | 88 | 4 | 88 | 0.308 |
| HELLER 2012^8^ | Glargine  (Once) | Degludec  (Once) | 16 | 157 | 58 | 472 | 1 |
| MATHIEU 2013^9^ | Glargine  (Once) | Degludec  (Once) | 16 | 161 | 21 | 165 | 0.5 |
| RASKIN 2000^10^ | Glargine  (Once) | NPH  (Once/twice) | 20 | 310 | 16 | 309 | 0.308 |
| RATNER 2000^11^ | Glargine  (Once) | NPH  (Once/twice) | 5 | 264 | 15 | 270 | 0.538 |
| HOME 2005^12^ | Glargine  (Once) | NPH  (Once/twice) | 31 | 292 | 44 | 293 | 0.538 |
| PIEBER 2000^13^ | Glargine  (Once) | NPH  (Once/twice) | 7 | 110 | 5 | 110 | 0.075 |
| RUSSELL JONES 2004^14^ | Detemir  (Once) | NPH  (Once) | 31 | 491 | 22 | 256 | 0.5 |
| HERMANSEN 2001^15^ | NPH  (Once) | Detemir  (Once/twice) | 7 | 56 | 4 | 57 | 0.115 |
| BARTLEY 2008^16^ | NPH  (Once/twice) | Detemir  (Once/twice) | 42 | 164 | 49 | 331 | 2 |

Abbreviations: N, number of patients with one or more events; NR, number randomised; PYs: person-years ^x^Time only included in cloglog model

- 1. Study data for severe/major hypoglycaemia- Poisson model

| Study | Comparator 1 | Comparator 2 | Trial follow-up time (years) | Comparator 1 | | | | Comparator 2 | | |
| --- | --- | --- | --- | --- | --- | --- | --- | --- | --- | --- |
|  |  |  |  |  |  |  |  | |  |  |
|  |  |  |  | NE | NR | PYs | N | | NR | PYs |
| CHATTERJEE 2007^1^ | NPH  (Twice) | Glargine  (Once) | 0.31 | 1 | 58 | 18 | 1 | | 58 | 18 |
| HERMANSEN 2004^2^ | NPH  (Twice) | Detemir  (Twice) | 0.35 | 45 | 297 | 103 | 40 | | 298 | 103 |
| HOME 2004^3^ | NPH  (Twice) | Detemir  (Twice) | 0.31 | 12 | 132 | 41 | 24 | | 139 | 43 |
| STANDL 2004^4^ | NPH  (Twice) | Detemir  (Twice) | 1.00 | 20 | 135 | 83 | 35 | | 154 | 146 |
| VAGUE 2003^5^ | NPH  (Twice) | Detemir  (Twice) | 0.50 | 41 | 146 | 29 | 56 | | 301 | 50 |
| KOLENDORF 2006^17^ | NPH  (Twice) | Detemir  (Twice) | 0.31 | 33 | 128 | 39 | 19 | | 125 | 38 |
| PIEBER 2005^18^ | NPH  (Twice) | Detemir  (Twice) | 0.23 | 5 | 129 | 30 | 6 | | 132 | 30 |
| HELLER 2012^8^ | Glargine  (Once) | Degludec  (Once) | 1.00 | 23 | 157 | 144 | 90 | | 472 | 429 |
| MATHIEU 2013^9^ | Glargine  (Once) | Degludec  (Once) | 0.50 | 40 | 161 | 81 | 33 | | 165 | 83 |
| FULCHER 2005^19^ | Glargine  (Once) | NPH  (Once) | 0.58 | 131 | 63 | 36 | 119 | | 65 | 38 |
| RASKIN 2000^10^ | Glargine  (Once) | NPH  (Once/twice) | 0.31 | 29 | 310 | 95 | 20 | | 309 | 95 |
| RATNER 2000^11^ | Glargine  (Once) | NPH  (Once/twice) | 0.54 | 11 | 264 | 142 | 24 | | 270 | 145 |
| HELLER 2009^20^ | Glargine  (Once) | Detemir  (Once/twice) | 1.00 | 53 | 144 | 133 | 146 | | 299 | 292 |
| RUSSELL JONES 2004^14^ | Detemir  (Once) | NPH  (Once) | 0.50 | 68 | 491 | 246 | 32 | | 256 | 128 |
| HERMANSEN 2001^15^ | NPH  (Once) | Detemir  (Once/twice) | 0.12 | 11 | 56 | 6 | 4 | | 57 | 7 |
| BARTLEY 2008^16^ | NPH  (Once/twice) | Detemir  (Once/twice) | 2.00 | 237 | 164 | 296 | 148 | | 331 | 740 |

Abbreviations: NE, number of events; NR, number randomised; PYs: person-years

- 1. Cloglog data used in shared parameter model

| - 1. Cloglog Data | | | | | | | |
| --- | --- | --- | --- | --- | --- | --- | --- |
| Study | Comparator 1 | Comparator 2 | Comparator 1 | | Comparator 2 | | Trial follow-up time (Years |
|  |  |  | N | NR | N | NR |  |
| LEEUW 2005^6^ | NPH  (Twice) | Detemir  (Twice) | 21 | 99 | 30 | 216 | 1 |
| RENARD 2011^7^ | Glargine  (Once) | Detemir  (Once) | 10 | 88 | 4 | 88 | 0.308 |
| HOME 2005^12^ | Glargine  (Once) | NPH  (Once/twice) | 31 | 292 | 44 | 293 | 0.538 |
| PIEBER 2000^13^ | Glargine  (Once) | NPH  (Once/twice) | 7 | 110 | 5 | 110 | 0.075 |

Information on mean age and HbA1c in each study is available in the NICE guideline on T1 Diabetes, Appendix G, page 383 section: G.4.2 Long-acting insulin, available here: https://www.nice.org.uk/guidance/ng17/evidence/appendix-g-pdf-435400239

- 1. References for data used in each model

1. Chatterjee S, Jarvis-Kay J, Rengarajan T, Lawrence IG, McNally PG, Davies MJ. Glargine versus NPH insulin: efficacy in comparison with insulin aspart in a basal–bolus regimen in type 1 diabetes--the glargine and aspart study (GLASS) a randomised cross-over study. Diabetes Research and Clinical Practice. 2007; 77(2):215-222
2. Hermansen K, Fontaine P, Kukolja KK, Peterkova V, Leth G, Gall MA. Insulin analogues (insulin detemir and insulin aspart) versus traditional human insulins (NPH insulin and regular human insulin) in basal–bolus therapy for patients with type 1 diabetes. Diabetologia. 2004; 47(4):622- 629
3. Home P, Bartley P, Russell-Jones D, Hanaire-Broutin H, Heeg JE, Abrams P et al. Insulin detemir offers improved glycemic control compared with NPH insulin in people with type 1 diabetes: a randomized clinical trial. Diabetes Care. 2004; 27(5):1081-1087
4. Standl E, Lang H, Roberts A. The 12-month efficacy and safety of insulin detemir and NPH insulin in basal–bolus therapy for the treatment of type 1 diabetes. Diabetes Technology and Therapeutics. 2004; 6(5):579-58
5. Vague P, Selam JL, Skeie S, Leeuw I, Elte JW, Haahr H et al. Insulin detemir is associated with more predictable glycemic control and reduced risk of hypoglycemia than NPH insulin in patients with type 1 diabetes on a basal–bolus regimen with premeal insulin aspart. Diabetes Care. 2003; 26(3):590-596
6. Leeuw I, Vague P, Selam JL, Skeie S, Lang H, Draeger E et al. Insulin detemir used in basal–bolus therapy in people with type 1 diabetes is associated with a lower risk of nocturnal hypoglycaemia and less weight gain over 12 months in comparison to NPH insulin. Diabetes Obesity and Metabolism. 2005; 7(1):73-82
7. Renard E, Dubois-Laforgue D, Guerci B. Non-inferiority of insulin glargine versus insulin detemir on blood glucose variability in type 1 diabetes patients: A multicenter, randomized, crossover study. Diabetes Technology and Therapeutics. 2011; 13(12):1213-1218
8. Heller S, Buse J, Fisher M, Garg S, Marre M, Merker L et al. Insulin degludec, an ultra-longacting basal insulin, versus insulin glargine in basal–bolus treatment with mealtime insulin aspart in type 1 diabetes (BEGIN Basal–bolus Type 1): a phase 3, randomised, open-label, treat-to-target non-inferiority trial. Lancet. 2012; 379(9825):1489-1497
9. Mathieu C, Hollander P, Miranda-Palma B, Cooper J, Franek E, Russell-Jones D et al. Efficacy and safety of insulin degludec in a flexible dosing regimen vs insulin glargine in patients with type 1 diabetes (BEGIN: Flex T1): a 26-week randomized, treat-to-target trial with a 26-week extension. Journal of Clinical Endocrinology and Metabolism. 2013; 98(3):1154-1162
10. Raskin P, Guthrie RA, Leiter L, Riis A, Jovanovic L. Use of insulin aspart, a fast-acting insulin analog, as the mealtime insulin in the management of patients with type 1 diabetes. Diabetes Care. 2000; 23(5):583-588
11. Ratner RE, Hirsch IB, Neifing JL, Garg SK, Mecca TE, Wilson CA. Less hypoglycemia with insulin glargine in intensive insulin therapy for type 1 diabetes. U.S. Study Group of Insulin Glargine in Type 1 Diabetes. Diabetes Care. 2000; 23(5):639-643
12. Home PD, Rosskamp R, Forjanic-Klapproth J, Dressler A. A randomized multicentre trial of insulin glargine compared with NPH insulin in people with type 1 diabetes. Diabetes/Metabolism Research and Reviews. 2005; 21(6):545-553
13. Pieber TR, Eugene-Jolchine I, Derobert E. Efficacy and safety of HOE 901 versus NPH insulin in patients with type 1 diabetes. The European Study Group of HOE 901 in type 1 diabetes. Diabetes Care. 2000; 23(2):157-162
14. Russell-Jones D, Simpson R, Hylleberg B, Draeger E, Bolinder J. Effects of QD insulin detemir or neutral protamine Hagedorn on blood glucose control in patients with type I diabetes mellitus using a basal–bolus regimen. Clinical Therapeutics. 2004; 26(5):724-736
15. Hermansen K, Madsbad S, Perrild H, Kristensen A, Axelsen M. Comparison of the soluble basal insulin analog insulin detemir with NPH insulin: A randomized open crossover trial in type 1 diabetic subjects on basal–bolus therapy. Diabetes Care. 2001; 24(2):296-301
16. Bartley PC, Bogoev M, Larsen J, Philotheou A. Long-term efficacy and safety of insulin detemir compared to Neutral Protamine Hagedorn insulin in patients with Type 1 diabetes using a treat-to-target basal–bolus regimen with insulin aspart at meals: a 2-year, randomized, controlled trial. Diabetic Medicine. 2008; 25(4):442-449
17. Kolendorf K, Ross GP, Pavlic-Renar I, Perriello G, Philotheou A, Jendle J et al. Insulin detemir lowers the risk of hypoglycaemia and provides more consistent plasma glucose levels compared with NPH insulin in Type 1 diabetes. Diabetic Medicine. 2006; 23(7):729-735
18. Pieber TR, Draeger E, Kristensen A, Grill V. Comparison of three multiple injection regimens for Type 1 diabetes: morning plus dinner or bedtime administration of insulin detemir vs. morning plus bedtime NPH insulin. Diabetic Medicine. 2005; 22(7):850-857
19. Fulcher GR, Gilbert RE, Yue DK. Glargine is superior to neutral protamine Hagedorn for improving glycated haemoglobin and fasting blood glucose levels during intensive insulin therapy. Internal Medicine Journal. 2005; 35(9):536-542
20. Heller S, Koenen C, Bode B. Comparison of insulin detemir and insulin glargine in a basal–bolus regimen, with insulin aspart as the mealtime insulin, in patients with type 1 diabetes: a 52- week, multinational, randomized, open-label, parallel-group, treat-to-target noninferiority trial. Clinical Therapeutics. 2009; 31(10):2086-2097
