# Supplementary material for: Different Methods for Modelling Severe Hypoglycaemic Events: Implications for Effectiveness, Costs and Health Utilities
Source: Pharmacoeconomics. 2018 Feb 14;36(5):523–32. doi: 10.1007/s40273-018-0612-y (PMC5906516; doi:10.1007/s40273-018-0612-y)
Supplement: Supplementary file 2 — Supplementary material 2 (DOCX 21 kb) [file 40273_2018_612_MOESM2_ESM.docx]

**Appendix B. Example WinBUGS code**

**#RE_Binomial model with logit link**

model{

for(i in 1:ns){

w[i,1] < -0

delta [i,t[i,1]] < -0

mu[i] ~ dnorm(0,.0001)

for (k in 1:na[i]) {

r[i,k] ~ dbin(p[i,t[i,k]],n[i,k]) logit(p[i,t[i,k]])<-mu[i] + delta[i,t[i,k]] rhat[i,k] <- p[i,t[i,k]] * n[i,k] dev[i,k] <- 2 * (r[i,k] * (log(r[i,k])-log(rhat[i,k])) + (n[i,k]- r[i,k]) * (log(n[i,k]-r[i,k]) - log(n[i,k]-rhat[i,k]))) }

resdev[i]<- sum(dev[i,1:na[i]])

for (k in 2:na[i]) { delta[i,t[i,k]] ~ dnorm(md[i,t[i,k]],taud[i,t[i,k]]) md[i,t[i,k]] <- d[t[i,k]] - d[t[i,1]] + sw[i,k] taud [i,t[i,k]] < -tau * 2 * (k - 1) / k

w[i,k] <- (delta[i,t[i,k]] - d[t[i,k]] + d[t[i,1]]) sw[i,k] <-sum(w[i,1:k-1])/(k-1) } }

d[1] <- 0

for (k in 2:nt){d[k] ~ dnorm(0,.0001) }

sd~dunif(0,2) tau<-1/pow(sd,2)

totresdev <- sum(resdev[])

A ~ dnorm(meanA,precA)

for (k in 1:nt) { logit(T[k]) <- A + (d[k] - d[1]) }

for (k in 1:nt) {Exp.cost[k] <- T[k] * cost }

for (k in 1:nt) {Exp.utility[k] <- T[k] *utility }

for (c in 1:(nt-1))

{ for (k in (c+1):nt) { lor[c,k] <- d[k] - d[c] log(or[c,k]) <- lor[c,k] }}

for (k in 1:nt) { rk[k]<-rank(d[],k) best[k]<-equals(rk[k],1)

}

}

**#RE Binomial model with cloglog link**

model{

for(i in 1:ns){

w[i,1] <- 0

delta[i,1] <- 0

mu[i] ~ dnorm(0,.0001)

for (k in 1:na[i]) { # LOOP THROUGH ARMS r[i,k] ~ dbin(p[i,k],n[i,k]) cloglog(p[i,k]) <- log(time[i]) + mu[i] + delta[i,k] rhat[i,k] <- p[i,k] * n[i,k]

dev[i,k] <- 2 * (r[i,k] * (log(r[i,k])-log(rhat[i,k]))+ (n[i,k]-r[i,k]) * (log(n[i,k]-r[i,k]) - log(n[i,k]-rhat[i,k]))) }

resdev[i] <- sum(dev[i,1:na[i]])

for (k in 2:na[i]) { delta[i,k] ~ dnorm(md[i,k],taud[i,k]) md[i,k] <- d[t[i,k]] - d[t[i,1]] + sw[i,k] taud[i,k] <- tau *2*(k-1)/k

w[i,k] <- (delta[i,k] - d[t[i,k]] + d[t[i,1]]) sw[i,k] <- sum(w[i,1:k-1])/(k-1) } }

totresdev <- sum(resdev[])

d[1]<-0

for (k in 2:nt){ d[k] ~ dnorm(0,.0001) }

sd ~ dunif(0,5)

tau <- pow(sd,-2)

A ~ dnorm(meanA,precA)

for (k in 1:nt) { cloglog(T[k]) <- log(timeA) + A + d[k] }

for (k in 1:nt) {Exp.cost[k] <- T[k] * cost }

for (k in 1:nt) {Exp.utility[k] <- T[k] * utility }

for (c in 1:(nt-1)) {

for (k in (c+1):nt) {

lhr[c,k] <- (d[k]-d[c])

log(hr[c,k]) <- lhr[c,k] }

}

for (k in 1:nt) {

rk[k] <- rank(d[],k) # assumes events are "bad"

best[k] <- equals(rk[k],1) #calculate probability that treat k is best

}}

**#RE Poisson model**

model{

for(i in 1:ns){

w[i,1] <- 0

delta[i,1] <- 0

mu[i] ~ dnorm(0,.0001)

for (k in 1:na[i]) {

r[i,k] ~ dpois(theta[i,k]) theta[i,k] <- lambda[i,k]*E[i,k] log(lambda[i,k]) <- mu[i] + delta[i,k] dev[i,k] <- 2*((theta[i,k]-r[i,k]) + r[i,k]*log(r[i,k]/theta[i,k])) }

resdev[i] <- sum(dev[i,1:na[i]])

for (k in 2:na[i]) { delta[i,k] ~ dnorm(md[i,k],taud[i,k]) md[i,k] <- d[t[i,k]] - d[t[i,1]] + sw[i,k] taud[i,k] <- tau *2*(k-1)/k w[i,k] <- (delta[i,k] - d[t[i,k]] + d[t[i,1]]) sw[i,k] <- sum(w[i,1:k-1])/(k-1) } }

totresdev <- sum(resdev[])

d[1]<-0

for (k in 2:nt){ d[k] ~ dnorm(0,.0001) }

sd ~ dunif(0,5)

tau <- pow(sd,-2)

A ~ dnorm(meanA,precA)

for (k in 1:nt) { log(T[k]) <- A + (d[k] -d[1]) }

for (k in 1:nt) { prob[k] <- 1- exp(-(T[k]) ) }

for (k in 1:nt) {Exp.cost[k] <- prob[k] * cost }

for (k in 1:nt) {Exp.utility[k] <- prob[k] * (totEvents *utility) }

for (c in 1:(nt-1)) {

for (k in (c+1):nt) {

lhr[c,k] <- (d[k]-d[c])

log(hr[c,k]) <- lhr[c,k]

}

}

for (k in 1:nt) {

rk[k] <- rank(d[],k)

best[k] <- equals(rk[k],1)

} }

**# RE Shared Parameter model**

model {

for(i in 1:NumStudiesC) { # indexes studies with cloglog data

mu[i] ~ dnorm(0, .0001)

delta[i,1] <- 0

w[i,1] <- 0

for (j in 1:na[i]) { # indexes arms

k[i,j] ~ dbin(p[i,j],n[i,j]) # binomial likelihood

cloglog(p[i,j]) <- log(time[i]/1) + mu[i] + delta[i,j]

rhat[i,j]<- p[i,j] * n[i,j]

dev[i,j]<- 2 * (k[i,j] * (log(k[i,j])-log(rhat[i,j])) + (n[i,j]-k[i,j]) * (log(n[i,j]-k[i,j]) - log(n[i,j]-rhat[i,j])))

}

for (j in 2:na[i]) { # indexes arms

delta[i,j] ~ dnorm(md[i,j],taud[i,j])

md[i,j] <- d[t[i,j]] - d[t[i,1]] + sw[i,j]

taud[i,j] <- tau *2*(j-1)/j

w[i,j] <- (delta[i,j] - d[t[i,j]] + d[t[i,1]])

sw[i,j] <- sum(w[i,1:j-1])/(j-1)

}

resdev[i] <- sum(dev[i,1:na[i]])

} # close study loop

for(i in 1:NumStudiesP) { # indexes studies with poisson data

mu[i + NumStudiesC] ~ dnorm(0, .0001)

delta[i + NumStudiesC,1] <- 0

w[i + NumStudiesC,1] <- 0

for (j in 1:naP[i]) { # indexes arms

r[i,j] ~ dpois(theta[i,j]) # Poisson likelihood

theta[i,j] <- lambda[i,j] * E[i,j] # failure rate * exposure

log(lambda[i,j]) <- mu[i + NumStudiesC] + delta[i + NumStudiesC,j]

dev[i + NumStudiesC,j] <- 2 * ((theta[i,j]-r[i,j]) + r[i,j] * log(r[i,j] / theta[i,j]))

}

for (j in 2:naP[i]) { # indexes arms

delta[i + NumStudiesC,j] ~ dnorm(md[i + NumStudiesC,j],taud[i + NumStudiesC,j])

md[i + NumStudiesC,j] <- d[tP[i,j]] - d[tP[i,1]]+ sw[i + NumStudiesC,j]

taud[i + NumStudiesC,j] <- tau *2*(j-1)/j

w[i + NumStudiesC,j]<- (delta[i + NumStudiesC,j] - d[tP[i,j]] + d[tP[i,1]])

sw[i + NumStudiesC,j] <- sum(w[i + NumStudiesC,1:j-1])/(j-1)

}

resdev[i + NumStudiesC] <- sum(dev[i + NumStudiesC,1:naP[i]])

} # close study loop

totresdev <- sum(resdev[])

d[1]<-0

for (j in 2:nt) d[j] ~ dnorm(0, .000}

sd ~ dunif(0,5) # vague prior for between-trial SD

tau <- pow(sd,-2) # between-trial precision = (1/between-trial variance)

for (c in 1:(nt-1)) {

for (j in (c+1):nt) {

lHR[c,j] <- d[j] - d[c]

log(HR[c,j]) <- lHR[c,j]

}

}

for (j in 1:nt) {

rk[j] <- nt+1-rank(d[],j)

best[j] <- equals(rk[j],1)

for (h in 1:nt) {

pRk[h,j] <- equals(rk[j],h

}

}}

**#Baseline RE model – Binomial with logit link**

model{

for (i in 1:ns){

r[i] ~ dbin(p[i],n[i])

logit(p[i]) <- mu[i]

mu[i] ~ dnorm(m,tau.m)

rhat[i] <- p[i] * n[i]

dev[i] <- 2 * (r[i] * (log(r[i])-log(rhat[i])) + (n[i]-r[i]) * (log(n[i]-r[i]) - log(n[i]-rhat[i])))

}

totresdev <- sum(dev[])

mu.new ~ dnorm(m,tau.m)

m ~ dnorm(0,.0001)

var.m <- 1/tau.m

tau.m <- pow(sd.m,-2)

sd.m ~ dunif(0,5)

logit(R) <- m

logit(R.new) <- mu.new

}

**#Baseline RE model – Binomial with Cloglog link**

model{

for( i in 1:ns) {

r[i] ~ dbin(p[i],n[i]) #Likelihood

cloglog(p[i]) <- b[i] + log(time[i]) #cloglog of response

b[i] ~ dnorm(d,prec) #Random effects model

rhat[i] <- p[i] * n[i] # expected value of the numerators

#Deviance contribution

dev[i] <- 2 * (r[i] * (log(r[i])-log(rhat[i]))

+ (n[i]-r[i]) * (log(n[i]-r[i]) - log(n[i]-rhat[i]))) }

b.new~dnorm(d,prec) #predictive dist. (log-odds)

resdev <- sum(dev[])

d ~ dnorm(0.0,1.0E-6) #vague prior for mean effect

cloglog(T1) <- b.new +log(1)

cloglog(T12) <- b.new +log(12)

sd ~ dunif(0,5) # uniform prior for RE st dev

prec <- pow(sd,-2)

}

**# Baseline RE model - Poisson**

model{

for(i in 1:ns){

r[i] ~ dpois(theta[i]) # Poisson likelihood

theta[i] <- lambda[i]*E[i] # failure rate * exposure

log(lambda[i]) <- mu[i] # model for linear predictor

mu[i] ~ dnorm(m,tau.m) # random effects model

#Deviance contribution

dev[i] <- 2*((theta[i]-r[i]) + r[i]*log(r[i]/theta[i])) }

# summed residual deviance contribution for this trial

totresdev <- sum(dev[]) #Total Residual Deviance

mu.new ~ dnorm(m, tau.m) # predictive distribution

m ~ dnorm(0,.0001) # vague prior for baseline effect

sd ~ dunif(0,5) # vague prior for between-trial SD

tau.m <- pow(sd,-2) # between-trial precision = (1/between-trial variance)

# Provide estimates of treatment effects T[k] on the natural (rate) scale

log(T) <- m } # *** PROGRAM ENDS
