# Supplementary material for: Different Methods for Modelling Severe Hypoglycaemic Events: Implications for Effectiveness, Costs and Health Utilities
Source: Pharmacoeconomics. 2018 Feb 14;36(5):523–32. doi: 10.1007/s40273-018-0612-y (PMC5906516; doi:10.1007/s40273-018-0612-y)
Supplement: Supplementary file 3 — Supplementary material 3 (DOCX 13 kb) [file 40273_2018_612_MOESM3_ESM.docx]

**Appendix C. Model fit and selection statistics**

Fixed effect and random effects models. For random effects models we also compared the fit of consistency and inconsistency models.

**Logit model: Convergence was assessed using two chains and was achieved by 40,000 simulations the random effects consistency model. Estimates are based on a further 80,000 updates.**

| **Model** | **No. of data points** | **Total Residual Deviance** | **Between-trials SD (posterior median) and 95% credible intervals** | **DIC** |
| --- | --- | --- | --- | --- |
| **Binomial RE consistency** | 32 | 32.72 | 0.27 (0.01, 0.75) | 198.59 |
| **Binomial RE inconsistency** | 32 | 33 | 0.29 (0.01. 0.82) | 199.81 |
| **Binomial FE consistency** | 32 | 35.25 | - | 197.66 |

**Clog-log model: Convergence was assessed using two chains and was achieved by 40,000 simulations the random effects consistency model. Estimates are based on a further 80,000 updates.**

| **Model** | **No. of data points** | **Total Residual Deviance** | **Between-trials SD (posterior median) and 95% credible intervals** | **DIC** |
| --- | --- | --- | --- | --- |
| **Cloglog RE consistency** | 32 | 32.64 | 0.27 (0.01, 0.73) | 198.41 |
| **Cloglog RE inconsistency** | 32 | 32.22 | 3.57 (1.7, 4.93) | 198.94 |
| **Cloglog FE inconsistency** | 32 | 35.26 | - | 197.60 |

**Poisson model: Convergence was assessed using two chains and was achieved by 50,000 simulations the random effects consistency model. Estimates are based on a further 50,000 updates.**

| **Model** | **No. of data points** | **Total Residual Deviance** | **Between-trials SD (posterior median) and 95% credible intervals** | **DIC** |
| --- | --- | --- | --- | --- |
| **Poisson RE consistency** | 32 | 32.13 | 0.6 (0.31, 1.13) | 226.08 |
| **Poisson RE inconsistency** | 32 | 33.23 | 0.43 (0.07, 1.03) | 226.76 |
| **Poisson FE consistency** | 32 | 71.56 | - | 258.61 |

**Shared parameter model: Convergence was assessed using two chains and was achieved by 25,000 simulations the random effects consistency model. Estimates are based on a further 100,000 updates.**

| **Model** | **No. of data points** | **Total Residual Deviance** | **Between-trials SD (posterior median) and 95% credible intervals** | **DIC** |
| --- | --- | --- | --- | --- |
| **Shared parameter RE consistency** | 40 | 40.02 | 0.51 (0.28, 0.88) | 274.89 |
| **Shared parameter RE inconsistency** | 40 | 42.06 | 0.31 (0.001, 0.73) | 276.14 |
| **Shared parameter FE consistency** | 40 | 85.81 | - | 311.26 |
